# Antibiotics and Opportunities of Their Alternatives in Pig Production: Mechanisms Through Modulating Intestinal Microbiota on Intestinal Health and Growth

**DOI:** 10.3390/antibiotics14030301

**Published:** 2025-03-14

**Authors:** Jung Yeol Sung, Zixiao Deng, Sung Woo Kim

**Affiliations:** Department of Animal Science, North Carolina State University, Raleigh, NC 27695, USA; jysung@ncsu.edu (J.Y.S.); zxdeng1222@gmail.com (Z.D.)

**Keywords:** antimicrobial growth promoter, bacteriophage, functional fatty acids, intestinal health, intestinal microbiota, growth, organic acid, phytobiotics, postbiotics, probiotics

## Abstract

Antibiotics at subtherapeutic levels have been used in pig diets as antimicrobial growth promoters. However, concerns about antibiotic resistance have increased the demand for alternatives to these antimicrobial growth promoters. This review paper explores the mechanisms through which antimicrobial growth promoters and their alternatives exert their antimicrobial effects. Additionally, this systemic review also covers how modulation of intestinal microbiota by antimicrobial growth promoters or their alternatives affects intestinal health and, subsequently, growth of pigs. The mechanisms and effects of antimicrobial growth promoters and their alternatives on intestinal microbiota, intestinal health, and growth are diverse and inconsistent. Therefore, pig producers should carefully assess which alternative is the most effective for optimizing both profitability and the health status of pigs in their production system.

## 1. Introduction

The first antibiotic discovered was penicillin by Alexander Fleming in 1928, and it has been used to treat bacterial infections in humans. In the 1940s, the positive effects of *Streptomyces aureofaciens* containing chlortetracycline residues on the growth performance of chicks were reported, indicating the potential use of subtherapeutic levels of antibiotics as antimicrobial growth promoters in farm animals [1,2]. Antimicrobial growth promoters have been used in pig diets to improve weight gain, feed efficiency, and reduce mortality since the 1950s. The use of antibiotics as antimicrobial growth promoters in pig diets is expected to increase weight gain by 4 to 16% and feed efficiency by 2 to 7% [2,3,4].

However, the prolonged use of antimicrobial growth promoters in pig diets over several decades has potentially contributed to antimicrobial resistance, which can threaten human health and production of animal proteins. First, the use of antimicrobial growth promoters in pig diets can increase antibiotic-resistant genes in pathogens and facilitate the direct transfer of these resistant genes from pigs to humans [5]. Antibiotic-resistant genes are more concentrated in the environment around pig farms that use antimicrobial growth promoters in pig diets compared with antibiotic-free farms [5]. Furthermore, *Acinetobacter baumannii* found in humans has evolved from being completely susceptible to antibiotics to becoming antibiotic-resistant by acquiring 45 resistance genes from the environment [6]. Additionally, antimicrobial resistance from pig production can increase the risk of transmitting antibiotic-resistant *Salmonella* through the food chain or to people working in the pig industry [7]. Antimicrobial resistance from the livestock industry may have contributed to *Salmonella* being one of the most common food-borne diseases [8]. The emergence of antimicrobial resistance reduces the effectiveness of antibiotics as the first-line therapy in humans, requiring the use of additional treatments. For instance, antibiotics may not be as effective in critical situations such as surgery, organ transplantation, and neonatal care, which can increase human mortality [9]. In 2019, approximately more than 2.8 million infections were resistant to antibiotics in the United States, leading to the deaths of 35,000 people [7]. The emergence of antimicrobial resistance would be more significant in developing countries compared with developed countries due to their limited capacity to develop new antibiotics [7].

**Figure 1:**
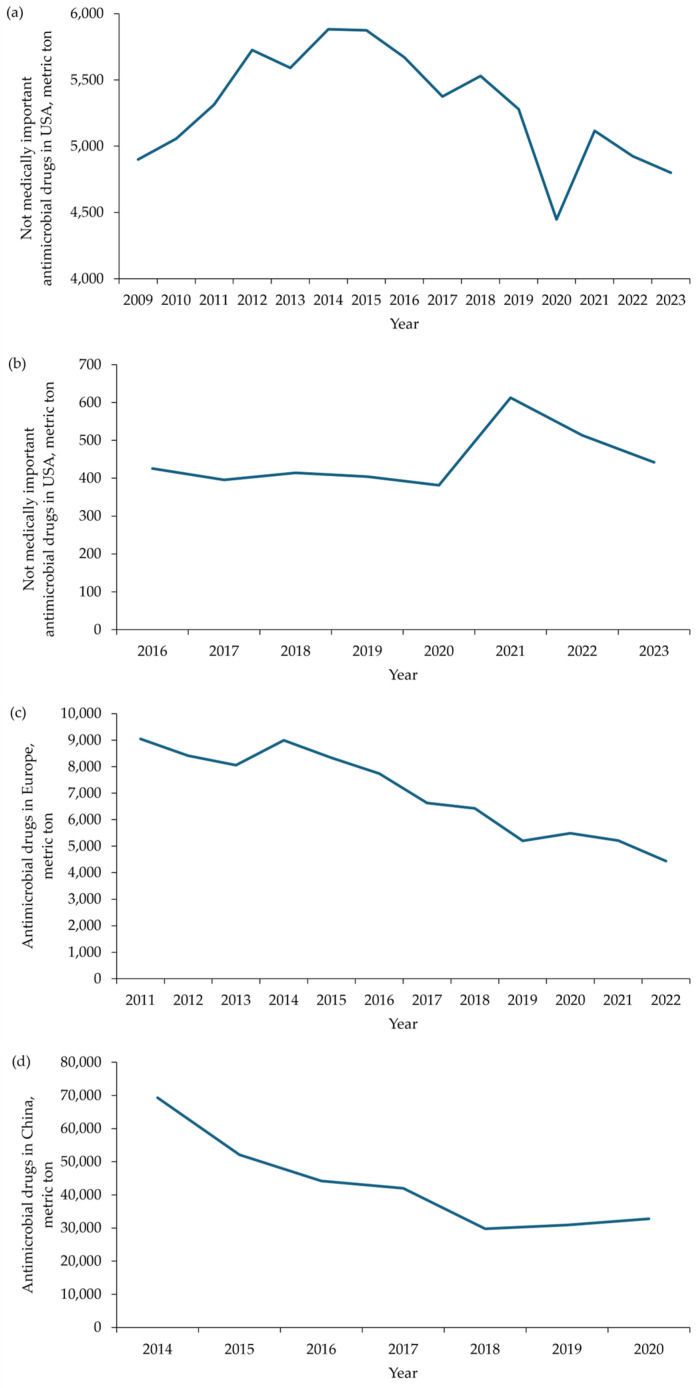
Domestic sales and distribution of not medically important antimicrobial drugs for use in (**a**) food-producing animals and (**b**) pigs in the United States and sales of antimicrobial drugs for use in food-producing animals in (**c**) Europe and (**d**) China.

Therefore, the amount of antimicrobial growth promoters in pig diets and water has decreased in recent years in the United States. Antibiotics are categorized as medically important and not medically important for humans [10]. Only antibiotics that are not medically important can be included in pig diets and water to promote growth performance. According to the summary report of FDA [11], domestic sales and distribution of ‘not medically important antimicrobial drugs’ for food-producing animals and pigs surged from 2020 to 2021 and have decreased between 2021 and 2023, indicating a recent reduction in their use for growth performance of food-producing animals and pigs (Figure 1a,b). Similarly, the total sales of antimicrobial drugs for food-producing animals in Europe significantly decreased by 28% between 2018 and 2022 (Figure 1c) [12]. The recent reduction in the use of antimicrobial drugs for food-producing animals in Europe is also attributed to enforced regulations [10,13]. Regulation (EU) 2019/6 prohibits the preventive use of antibiotics in groups of animals and medicated feeds, mandates the monitoring of veterinary medicinal products including antibiotics, and requires clear justification for the use of antimicrobials in disease prevention among animals. The total sales of antimicrobial drugs for food-producing animals in China significantly decreased by 53% between 2014 to 2020 (Figure 1d) [14]. The recent decline in antibiotic use for food-producing animals in China results from efforts by the Chinese Ministry of Agriculture and Rural Affairs to regulate excessive antibiotic use [15]. In Brazil, the use of antibiotics in pig production is reducing, which is partially supported by recent survey. Specifically, the use of antibiotics in 25 pig farms in Brazil was recorded in 2016 and 2020 [16]. In 2016, 74% of pigs were exposed to antibiotics during their lifetime, but this decreased to 41% in 2020. According to Tiseo et al. [17], Asia had the highest antibiotic use for food-producing animals at 57,167 tons, followed by South America at 14,500 tons, Europe at 9000 tons, North America at 7200 tons, and Africa at 4606 tons. Although antibiotic use for food-producing animals in Africa was 4606 tons in 2017, and by 2030, it is expected to increase by 37%—the highest increase among all continents [17].

Additionally, consumer perceptions of antibiotic use in food-producing animal feeds are contributing to its decline, as shown by a recent review in which 77% of 124 studies reported significant consumer concerns about antibiotics in animal feeds [18].

However, addressing issues in pig production (e.g., post-weaning diarrhea, heat stress, overstocking, etc.) has become more challenging due to the increasing severity of these problems in antibiotic-free production systems [19]. For this reason, extensive research on alternatives to antimicrobial growth promoters has been conducted to identify effective options including probiotics [20,21,22], prebiotics [23,24], postbiotics [25,26,27,28,29], phytobiotics [30,31,32,33,34], organic acids [35,36], bacteriophages [37,38], etc. To identify potent alternatives, it is important to understand the mechanisms of how antimicrobial growth promoters in pig diets can improve intestinal health and growth performance of pigs. Antimicrobial growth promoters have several mechanisms, but their positive effects on growth performance of animals are primarily attributed to modulation of intestinal microbiota and intestinal health [39]. The modulation of intestinal microbiota is important because the interaction between intestinal microbiota and the host affects intestinal immune responses and nutrient digestion/absorption, which can potentially influence growth performance of pigs [40]. Likewise, alternatives to antimicrobial growth promoters potentially modulate intestinal microbiota and intestinal health and improve growth performance. However, the impact and mechanisms on intestinal microbiota, intestinal health, and growth performance may be different between antimicrobial growth promoters and their alternatives [41]. Therefore, the objective of this review is to understand the mechanisms of antimicrobial growth promoters and their alternatives on intestinal microbiota, characterize how they shape intestinal microbiota, and explore the potential impacts of shifted intestinal microbiota on intestinal health and growth of pigs.

## 2. Mechanisms of Antibiotics

### 2.1. Inhibition of Cell Wall Synthesis

**Figure 2:**
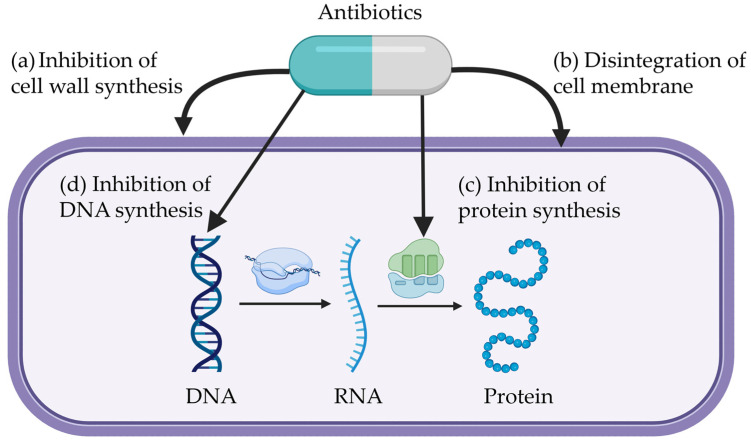
Mechanisms of antibiotics. Antibiotics inhibit growth of bacteria through the following mechanisms: (**a**) inhibition of cell wall synthesis (e.g., fosfomycin, bacitracin, and penicillin) [45,46], (**b**) disintegration of cell membrane (e.g., monensin, salinomycin, and narasin) [52,53], (**c**) inhibition of protein synthesis (e.g., neomycin, tetracycline, and chloramphenicol) [48,57], (**d**) inhibition of DNA synthesis (e.g., rifamycin and carbadox) [66]. The figure was generated by the authors based on concepts modified from Gaskins et al. [67], Broom [68], and Uddin et al. [69].

Bacterial cell walls provide structural integrity and protect cell membranes. Therefore, the biosynthesis of cell walls is important for the survival of bacteria [42]. The key component of bacterial cell walls is the peptidoglycan backbone, which imparts rigidity to the cell wall. Bacterial cell wall synthesis occurs in three stages: (1) the cytoplasmic stage, (2) the membrane-associated stage, and (3) the extra-cytoplasmic stage [43]. Antibiotics can inhibit bacterial cell wall synthesis by interfering with each stage of the process (Figure 2a; Table 1). When bacterial cell wall synthesis is inhibited, bacteria are unable to maintain the shape, which leads to cell rupture [44].

During the cytoplasmic stage, peptides of cell wall are synthesized using specific amino acids (gram-positive bacteria: Ala-Glu-Lys-Ala-Ala; gram-negative bacteria: Ala-Glu-diaminopimelic acid-Ala-Ala) [43]. Those peptides are building blocks for peptidoglycans. The synthesis of these pentapeptides involves the action of various enzymes (e.g., mur enzymes, alanine racemase, D-alanyl-D-alanine ligase, etc.). Fosfomycin and seromycin are typical antibiotics that inhibit the first stage. Fosfomycin inhibits Mur enzyme activity, whereas seromycin is an inhibitor of alanine racemase and D-alanyl-D-alanine ligase, preventing the first stage of bacterial cell wall synthesis [45,46].

During the membrane-associated stage, precursors of lipid intermediate are formed, which will serve as components and carriers for the peptidoglycan precursors across the bacterial membrane [43]. The targets of antibiotics that inhibit the membrane-associated stage are MraY and lipid molecules (e.g., undecaprenyl pyrophosphate, lipid II, etc.). MraY is an enzyme that transfers a peptidoglycan precursor onto lipid carriers and undecaprenyl pyrophosphate is an example of lipid carriers [47]. Lipid II is a subunit of peptidoglycan precursors. Tunicamycin, liposidomycin, and mureidomycin are antibiotics that inhibit the activity of MraY. Bacitracin inhibits the dephosphorylation of undecaprenyl pyrophosphate, thereby interfering with its recycling [48]. Lipid II is inactivated by binding to antibiotics including mannopeptimycin, lantibiotic, defensin, and glycopeptide antibiotics [49].

Peptidoglycan chains are cross-linked during the extra-cytoplasmic stage [43]. This step is modulated by enzymes outside of the cytoplasm (transpeptidase, endopeptidase, carboxypeptidase, and transglycosylase), which are associated with penicillin-binding proteins. Beta-lactam antibiotics (e.g., penicillin, cephalosporin, cephamycin, etc.) inactivate these enzymes by binding to their respective penicillin-binding proteins [50].

### 2.2. Disintegration of Cell Membrane

Disintegration of bacterial cell membranes leads to cell death due to the loss of shape control and the cessation of DNA and protein synthesis [51]. The majority of cell membrane-targeting antibiotics bind to phospholipids in the cytoplasmic cell membrane, leading to membrane aggregation (Figure 2b; Table 1) [52]. This interaction creates pores that disrupt the cytoplasmic membrane’s integrity, resulting in the leakage of ions and proteins from the cell. As a result, the loss of cellular components impairs membrane potential and overall cellular function. Daptomycin and ionophore including monensin, salinomycin, and narasin target bacterial cytoplasmic membranes [52,53].

Both gram-positive and gram-negative bacteria have a cytoplasmic membrane, but only gram-negative bacteria are protected by an additional outer membrane rich in lipopolysaccharide [54]. However, the outer membrane is more permeable compared with cytoplasmic membrane due to the presence of porins in the outer membrane [51]. Nisin is an antibiotic that binds to lipid II in the bacterial membrane and forms pores that disrupt membrane integrity [55].

### 2.3. Inhibition of Protein Synthesis

Translation of mRNA into proteins occurs at the ribosome level. Unlike eukaryotic cells, bacterial cells have ribosomes composed of a small (30S) and a large (50S) subunit [56]. A tRNA molecule binds to a specific site (aminoacyl, peptidyl, and exit site) of ribosome during initiation, elongation, and termination steps of translation process. Antibiotics that inhibit bacterial protein synthesis mainly target three essential components of bacterial ribosomes: (1) the aminoacyl site on the 30S subunit, (2) the peptidyl transferase center on the 50S subunit, and (3) the exit site on the 50S subunit (Figure 2c; Table 1) [57].

Antibiotics targeting the aminoacyl site on the 30S subunit are aminoglycoside families including neomycin, kanamycin, and puromycin [48,57]. After binding to the 30S subunit, these antibiotics inhibit codon recognition and translocation, leading to incorrect tRNA recognition by rRNA [58]. Similarly, tetracycline binds near the aminoacyl site on the 30S subunit, not directly at the site, and prevents the binding of tRNA to the aminoacyl site [59]. The peptidyl transferase center on the 50S subunit is the place where peptide bond formation occurs. Antibiotics such as chloramphenicol, clindamycin, sparsomycin, streptogramin, and oxazolidinone inhibit tRNA binding to its substrate or prevent peptide formation by directly binding to the peptidyl transferase center [60]. Finally, when peptides are further elongated, they should exit through the exit site on the 50S subunit. Macrolide antibiotics block the pathway to the elongating peptide’s exit tunnel, causing the premature release of peptidyl-tRNA intermediates and inhibiting protein synthesis [61].

### 2.4. Inhibition of DNA Synthesis

Bacterial DNA synthesis can be inhibited by blocking RNA transcription and synthesis. RNA polymerase is the enzyme responsible for synthesizing RNA from a DNA template (Figure 2d; Table 1) [62]. Rifamycin is an antibiotic that binds to bacterial RNA polymerase, thereby inhibiting RNA transcription [63]. Rifamycin also interferes with RNA synthesis by suppressing elongation of RNA.

To synthesize DNA, Type II topoisomerases (DNA gyrase and topoisomerase IV) are required to cut strands of DNA helix for DNA tangles and supercoils [64]. Quinoxalines are bacterial type II topoisomerase inhibitors that prevent the cleavage and rejoining of DNA strands [65]. Bacteria exposed to quinoxalines are unable to maintain the topological state of their DNA, resulting in the cessation of DNA synthesis [66]. Olaquindox, mequindox, quincetone, cyadox, and carbadox are examples of quinoxaline families.

### 2.5. Antimicrobial Resistance

However, bacteria can develop antimicrobial resistance through mutations and selections or gene transfer [70]. Mutations and selections of genes enable bacteria to acquire genes that encode enzymes capable of hydrolyzing antibiotics (e.g., β-lactamases), develop efflux pumps that expel antibiotics from cells, or modify antibiotic binding sites in bacterial cell walls [71]. Mutations in a single gene only slightly enhance bacterial survival against antibiotics. However, these mutations remain significant because once bacteria withstand antibiotic exposure, they can acquire additional mutations or genetic material, ultimately leading to complete antibiotic resistance [72]. In contrast, bacteria can horizontally transfer their antibiotic-resistant genes to antibiotic-sensitive bacteria. Horizontal gene transfer occurs in three distinct steps: transformation, conjugation, and transduction [73].

## 3. Mechanisms of Alternatives to Antimicrobial Growth Promoters

In this paper, we selected and reviewed the mechanisms by which organic acid, phytobiotics, probiotics, and other potentials (postbiotics, functional fatty acid, and bacteriophage) exhibit antimicrobial properties. The first three additives have been traditionally used in pig diets as alternatives to antimicrobial growth promoters, supported by extensive research in the literature [74,75,76,77,78,79]. The other additives are mostly not used in pig diets except for postbiotics [79]. However, these alternatives have the potential to replace antimicrobial growth promoters due to their distinct mechanisms and the opportunity for improvement through novel technologies.

### 3.1. Organic Acid

Organic acids are commonly used to alleviate the adverse effects associated with the limited digestive and absorptive capacity of pigs after weaning [40]. This period is characterized by a transition from sow milk of sows to solid diets, an immature digestive system, and various external stressors [80]. Organic acids support the host in digesting proteins more efficiently by reducing gastric pH and facilitating the conversion of inactive pepsinogen into active pepsin [76]. Several types of organic acids are widely used as feed additives in pig production, such as citric acid [81,82], benzoic acid [35], fumaric acid [83], formic acid [84], lactic acid [85], and propionic acid [86].

**Figure 3:**
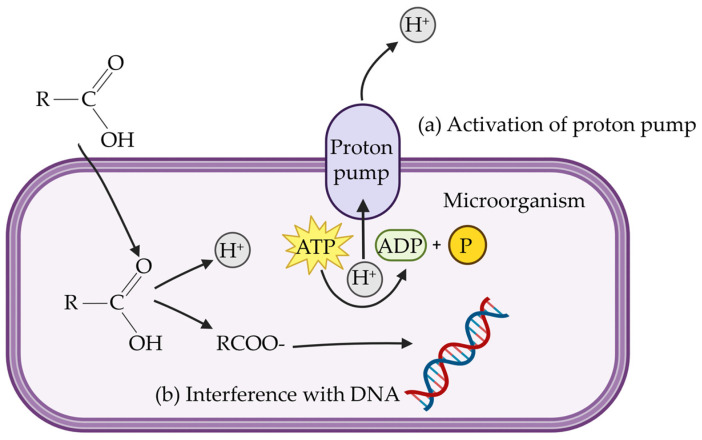
Mechanisms of organic acid. Organic acids show antimicrobial effects through (**a**) lowering cytoplasmic pH by activation of proton pump [90] and (**b**) interference with DNA [91,92].

Antimicrobial effects are a key function of organic acids. The pKa value of organic acid is considered an important factor in influencing its mechanisms [87]. The pKa of an acid is a measure of its acid strength, defined as the negative logarithm of the acid dissociation constant Ka [88]. Typically, more undissociated organic acids can exist in a lower pH environment. Organic acids can dissociate and release H⁺ ions as environmental pH increases [89]. Therefore, the mechanisms of organic acids are influenced by the environmental pH and their pKa values. In their undissociated form, organic acids can diffuse across bacterial cell membranes. Once they dissociate inside the cell, they release H⁺ ions, thereby lowering the intracellular pH. Most bacterial species are unable to grow or survive in an extremely acidic environment. To counteract the drop in pH, microorganisms activate proton pumps, which consume energy (Figure 3a) [90]. Meanwhile, the anion (RCOO⁻) interferes with DNA replication, disrupts metabolic processes, and contributes to increased osmotic pressure within the cell, ultimately compromising bacterial viability (Figure 3b) [91,92]. Both mechanisms contribute to antimicrobial effects of organic acids.

Organic acids have been used as an alternative to antimicrobial growth promoters because of their prominent antimicrobial effect. However, different types of organic acids have different efficiency on microorganisms [93]. The differences in antimicrobial capacity of organic acids are mainly attributed to: (1) ability to pass through the microorganism cell wall [94] and (2) functions of dissociated anions [95]. In addition, protected organic acids have been used in pig diets for decades, such as coating and microencapsulation. These technologies improve stability, reduce the order of organic acids [96], and allow them to slowly release in the lower intestinal tract of animals [97]. Recently, mixtures of organic acids are receiving more attention and indicating that the mixture could be more effective compared with individual organic acids because of synergistic effects of different pKa values and more broad-spectrum activity [95].

### 3.2. Phytobiotics

Phytobiotics are considered biologically active compounds derived from plants including seeds, leaves, and roots [77]. Phytobiotics can generally be classified into four main categories: (1) herbs, (2) spices, (3) essential oils, and (4) oleoresins [98,99]. Antimicrobial effects of phytobiotics are attributed to their key components including phenolics, organosulfur compounds, terpenes, or aldehydes [31,99].

**Figure 4:**
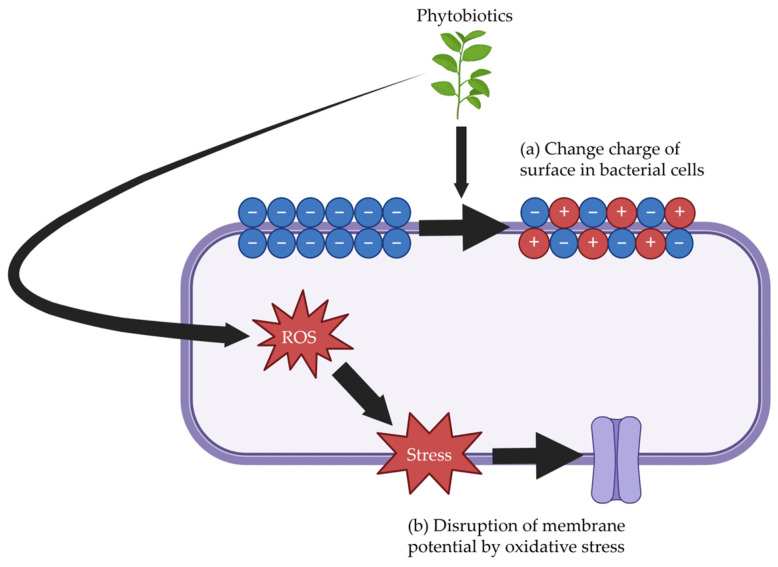
Mechanisms of phytobiotics. Phytobiotics show antimicrobial effects through (**a**) change in charge of surface in bacterial cells [100] and (**b**) disruption of membrane potential by oxidative stress [102].

Phytobiotics have distinct mechanisms of antimicrobial effects. First, phytobiotics have the potential to change the charge of surfaces in bacterial cells, which leads to damage to the cells (Figure 4a) [100]. Specifically, the surface of bacterial cells has a negative charge due to the presence of carboxyl and phosphate groups in the membrane. However, phytobiotics reduce the negative charge on the surface of bacterial cells, leading to cell dysfunction and damage [101]. Second, phytobiotics induce the production of reactive oxygen species in bacteria, which causes oxidative stress (Figure 4b) [102]. Glutathione protects bacterial cells against oxidative stress induced by phytobiotics [103]. However, phytobiotics deplete bacterial glutathione, leading to oxidative stress that alters membrane potential, disrupts barriers, and induces cell death.

Phytobiotics promote Nrf2-ARE pathway which regulates the expression of antioxidants and detoxifying enzymes including superoxide dismutase, catalase, and glutathione peroxidase [104]. As a result, phytobiotics protect the intestinal tract from oxidative stress. Furthermore, phytobiotics potentially inhibit quorum sensing which allows bacteria to communicate with each other [105,106,107]. Quorum sensing is crucial for the spread of virulence factors in enteric pathogens within the intestinal tract [108]. Phytobiotics inhibit quorum sensing by blocking signaling molecules including acyl-homoserine lactones small polypeptides and autoinducer-2 [109].

However, there are several challenges to using phytobiotics in pig diets as alternatives to antimicrobial growth promoters. Some phytobiotics can reduce feed intake in pigs due to their distinct odors and flavors, which may mask their potential beneficial effects on intestinal microbiota and intestinal health of pigs [77]. Additionally, the efficacy of phytobiotics is highly variable and depends on factors such as the specific components of phytobiotics, plant sources, and extraction methods [98]. Finally, phytobiotics may be quickly absorbed in the small intestine, reducing their ability to interact with intestinal microbiota [110].

### 3.3. Probiotics

Probiotics are live microbes that beneficially affect the host animal by improving its intestinal microbial balance [111]. The common probiotics used in pig diets include *Lactobacillus*, *bifidobacterium*, *Bacillus*, *Enterococcus*, etc. Probiotics is considered another alternative to antimicrobial growth promoters because of their beneficial effects, such as disease prevention, immune modulation, and restoration of microbial equilibrium [112].

**Figure 5:**
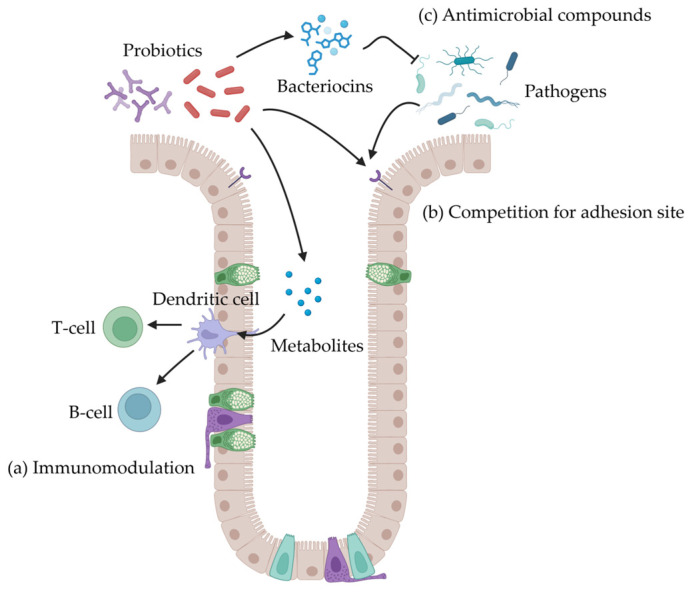
Mechanisms of probiotics. Probiotics show antimicrobial effects through (**a**) immunomodulation, such as innate and acquired immune system [114,115], (**b**) competition for adhesion site [119,120], and (**c**) secretion of antimicrobial compounds [124,125].

Probiotics have different mechanisms as an alternative to antimicrobial growth promoters [113]. First, probiotics could modulate the immune system, including innate and acquired immune systems. The metabolites, cell wall components, and DNA produced by probiotics can directly or indirectly influence the immune cells (Figure 5a) [114,115,116]. Furthermore, probiotics pass through intestinal epithelial cells and M cells on Peyer’s patches strengthening innate and acquired mucosal immunity [117]. Additionally, probiotics enhance the cytotoxic activity of NK cells and the phagocytic function of macrophages [118]. Specifically, probiotics activate nitric oxide synthase in macrophages leading to death of bacterial cells. Second, probiotics have a direct effect on other intestinal microorganisms, including commensal or pathogenic microorganisms. The competition for adhesion sites is one of methods to reduce the pathogenic microorganism colonization [119,120]. Probiotics prevent lipopolysaccharide from binding to the CD14 receptor, inhibiting NF-κB activation and suppressing proinflammatory cytokine production [121]. In addition to the inhibition of NF-κB, Probiotics suppress the activator protein 1 (AP-1) transcription factor by inhibiting C-Jun, which is regulated through the Mitogen-activated protein kinase pathway, which decreases inflammation in mucosa [122]. Moreover, the survival of microbial populations depends on their ability to compete for nutrients and energy within their environment. Probiotic microorganisms can consume nutrients that are essential for maintenance and growth of pathogens (Figure 5b) [123]. Third, probiotics can produce antimicrobial compounds to modify the intestinal microbiota. Bacteriocins, such as lactic acid, and deconjugated bile acids show a strong antimicrobial activity (Figure 5c) [124,125].

Even though probiotics are known to improve intestinal health of animals, attention is needed to ensure the transfer of probiotic microorganisms from pig diets to the intestinal tract. The gastric acid, bile acid, and digestive enzymes can significantly affect the colonization of probiotic microorganisms [126]. In addition, maintaining a stable and high level of probiotic microorganisms in the gastrointestinal tract requires frequent dosing over a specified period. This means reducing the interval between doses may be necessary to achieve optimal results [127]. The indigenous bacteria in the gastrointestinal tract of the host can be another factor that influences the probiotic effects due to the symbiotic relationship with them [128]. Therefore, the effects of probiotics usually differ from one individual to another. For pigs, weaning period is a key window to modulate the intestinal microbiota population with probiotics because of remarkable changes during the period [129].

### 3.4. Other Potentials: Postbiotics, Functional Fatty Acid, and Bacteriophage

Postbiotics include non-living microorganisms and their cell walls (e.g., peptidoglycan, lipoteichoic acid) and metabolites (e.g., bacteriocin, exopolysaccharide) [130]. Postbiotics exert antimicrobial effects through various mechanisms. Postbiotics can compete with pathogens for binding to the mucosa and epithelium in the intestinal tract, potentially reducing the activation of immune responses [131]. Postbiotics can also bind directly to receptors on bacteria, inactivating pathogenic strains such as *Clostridia* and *E. coli* [132]. Moreover, cellular components or secondary metabolites of postbiotics (e.g., lactic acids and bacteriocins) can neutralize bacteria by diffusing across bacterial membranes [133,134]. The typical postbiotics used in pig diets are derived from yeast and bacteria. Yeast cell walls contain mannan-oligosaccharides, β-glucans, and β-D-glucans, which can positively modulate immune systems [29]. Furthermore, mannan-oligosaccharides and β-glucans in yeast cell walls have prebiotic effects, which suppress the activation of NF-κB and oxidative stress in the intestinal tract of pigs [28]. Furthermore, *Lactobacillus* postbiotics are widely used for their ability to suppress intestinal inflammation through peptidoglycans in postbiotics [135]. Specifically, peptidoglycans enhance the concentrations of inducible nitric oxide synthase and cyclooxygenase-2, which partially contribute to anti-inflammatory effects [136]. Additionally, peptidoglycans are recognized by nucleotide-binding oligomerization domain receptors, including nucleotide-binding oligomerization domain 1, which plays a key role in activating the innate immune system [137].Functional fatty acids include medium-chain (6 to 12 carbons) and long-chain fatty acids (more than 12 carbons). Caproic acid, caprylic acid, capric acid, and lauric acid are typical examples of medium-chain fatty acids, whereas myristic acid, eicosapentaenoic acid, docosahexaenoic acid, and arachidonic acid are considered typical long-chain fatty acids [138,139,140]. The antimicrobial effects of medium-chain fatty acids are attributed to their bacteriostatic (inhibition of bacterial growth) and bactericidal properties (killing bacteria), as they can penetrate bacterial cells, disrupt cell membranes, alter cytosolic enzymes, and damage cells [141,142]. In contrast to organic acids, which are often used to reduce intestinal pH and inhibit pathogen colonization, the antimicrobial effects of medium-chain fatty acids primarily occur through the disruption of phospholipid membranes [143]. The antimicrobial effects of medium-chain fatty acids are more pronounced in gram-positive bacteria compared with gram-negative bacteria because gram-positive bacteria have simpler membrane structures. In addition, acquiring resistance to medium-chain fatty acids is challenging for pathogenic bacteria, making them more susceptible to these acids [144]. Some long-chain fatty acids such as myristic acid can directly bind to the cell wall of pathogenic bacteria and induce death of the cells through altering membrane permeability [145]. Furthermore, long-chain fatty acids can kill bacteria by disrupting electron transport [146], uncoupling oxidative phosphorylation [147], causing cell lysis [148], inhibiting enzyme activity [149], or blocking nutrient uptake [150].

Bacteriophage is a virus that infects bacterial cells [151]. Unlike other alternatives to antimicrobial growth promoters, most bacteriophages have host specificity. For example, a single bacteriophage targets only *Salmonella* or *E. coli* [152]. For this reason, bacteriophage cocktails containing specific bacteriophages are widely used to cover various pathogenic bacteria rather than individual bacteriophages [38,153]. Bacteriophages have two strategies for killing bacteria: lytic and lysogenic. When lytic bacteriophages attach to bacterial cells, their genetic information enters the bacterial cytoplasm [154]. The ribosomes of bacteria then synthesize progeny phages. When the cells die, the replicated bacteriophages are released, which can potentially infect other bacteria [155]. Lysogenic bacteriophages also bind to bacterial cells and provide their viral DNA into cytoplasm. However, bacteriophage genome integrates into the bacterial chromosome and is replicated (prophage) [156]. As a result, prophage is transferred to daughter bacterial cells. The transfer of the viral genome also occurs through horizontal gene transfer [157]. A prophage can potentially be converted into a lytic phage, which can directly kill bacterial cells.

## 4. Impact of Antimicrobial Growth Promoters on Intestinal Microbiota, Intestinal Health, and Growth of Pigs

The mechanisms underlying the supplementation of antimicrobial growth promoters in pig diets to improve growth performance remain unclear and cannot be attributed to a single factor. However, the mechanisms of antimicrobial growth promoters or their alternatives are likely related to their impact on the modulation of intestinal microbiota and intestinal health [67]. This hypothesis is supported by the results of previous studies reporting that feeding antimicrobial growth promoters improved growth performance of normal animals, but not of germ-free animals [158,159]. The modulation of intestinal microbiota has the potential to enhance intestinal health and, subsequently, improve the growth performance of pigs. In this review, we provided a summary of recent research on bacitracin and carbadox, which are commonly used antimicrobial growth promoters in pig diets (Table 1 and Table 2) [160,161]. Peer-reviewed papers on bacitracin and carbadox were searched from Scopus using the keywords ‘pig’ and ‘bacitracin’ ‘carbadox’ or ‘antibiotics’ for systemic review. Other antimicrobial growth promoters have also been widely used in pig diets, including chlortetracycline, penicillin, neomycin, oxytetracycline, and tylosin [162,163]. However, these antibiotics should be prescribed and technically should not be used in pig diets to promote growth performance because they are classified as medically important [10,164]. In contrast, bacitracin and carbadox are classified as ‘not medically important’, which can be legitimately used in pig diets to promote growth and feed efficiency of pigs [164]. Bacitracin and carbadox primarily target gram-positive bacteria [48,165]. However, bacitracin and carbadox may directly target some gram-negative bacteria or indirectly affect them by modulating the activity of gram-positive bacteria in the intestinal tract [26]. Supplementation of bacitracin reduced the relative abundance of potentially pathogenic gram-positive bacteria including *Streptococcus* (potentially causing diarrhea) [166], *Clostridium sensu stricto* (opportunistic pathogen causing inflammation) [167], [*Ruminococcus*] *gauvreauii*, and *Ruminococcus UCG-005* (causing chronic inflammation and diarrhea) [168,169] in digesta or feces of unchallenged pigs (Table 2). Additionally, carbadox decreased the abundance of *Slackia* (causing diarrhea) [170], *Catenibacterium* (causing obesity) [171], and *Streptococcus* (potentially causing diarrhea) [166] in pig feces (Table 3). Suppression of these potentially pathogenic bacteria could help reduce subclinical immune responses, saving energy and nutrients for growth performance that would otherwise be used for metabolic cost to activate immune responses [68]. Furthermore, bacitracin and carbadox were also effective in modulating intestinal microbiota when pigs were challenged with enterotoxigenic *E. coli.* Bacitracin or carbadox decreased the abundance of pathogenic bacteria including *Streptococcus* (potentially causing diarrhea) [166], *Escherichia-Shigella* (causing diarrhea) [170], and *Dorea* (causing enteric disease) [172], whereas increased the abundance of beneficial bacteria such as *Bifidobacterium* (probiotic effect) [173], *Propionibacteriaceae* (contributing to epithelial cell development and mucus production) [174], *Lactobacillaceae* (positively modulating immune system) [26], and *Blautia* (probiotic effects) in the intestinal tract (Table 1 and Table 2) [175]. The effects of bacitracin on growth performance were more pronounced in pigs challenged with enterotoxigenic *E. coli* compared with non-challenged pigs because the negative impact of an acute enteric challenge is greater than that of a subclinical challenge [176]. The majority of previous studies on bacitracin and carbadox have focused on modulation of microbiota in digesta or feces of pigs (Table 1 and Table 2). However, Xu et al. [25] and Duarte et al. [80] reported that bacitracin increased the relative abundance of beneficial bacteria in the jejunal mucosa-associated microbiota.

**Figure 6:**
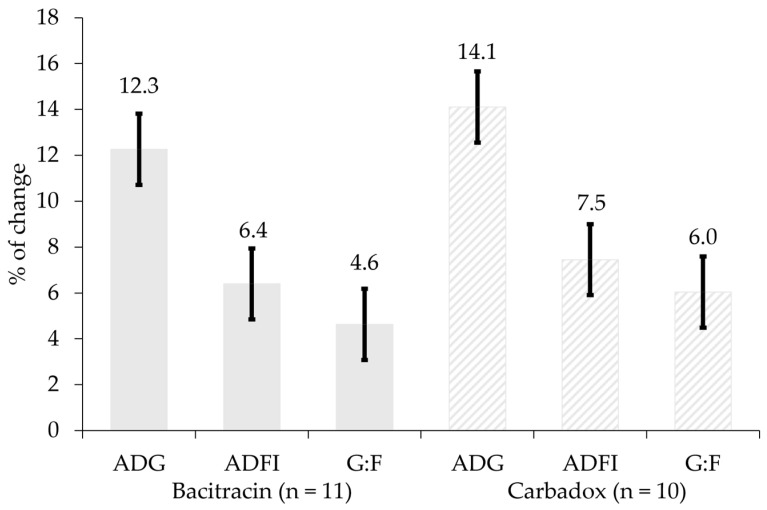
Changes in the average daily gain (ADG), average daily feed intake (ADFI), and gain to feed (G:F) of nursery pigs by bacitracin or carbadox in pig diets. The selected studies for bacitracin were Ángel-Isaza et al. [199], Choi et al. [35], Duarte et al. [80], Duarte et al. [26,80], Franco et al. [208], Han et al. [209], Hung et al. [168], Lin and Yu [197,198], Tian and Piao [210], and Xu et al. [25]. The selected studies for carbadox were Dahmer and Jones [211], Dahmer et al. [212], He et al. [177], Jinno et al. [207], Kim et al. [203,206], Kommera et al. [32], Outlaw et al. [213], and Wilt and Carlson [214]. Error bars represent standard error.

When the intestinal tract is infected by exogenous pathogens subclinically, activation of the immune system and tissue damage in the intestines occur [21,80]. However, activating the immune system and repairing damaged tissues in the intestines require energy and nutrients, potentially diverting resources that could otherwise be used to promote growth performance of pigs [68]. Intestinal commensal microorganisms provide defense against pathogens by modulating immune status in the intestinal tract. When pigs are enterically challenged with exogenous pathogens, dysbiosis of intestinal microbiota occurs, leading to an increased proliferation of pathogenic bacteria [40]. Antimicrobial growth promoters in pig diets potentially suppress excessive inflammation by modulating intestinal microbiota, which inhibits or mitigates subclinical or clinical immune responses in the intestinal tract [26,177]. For example, the supplementation of bacitracin decreased the expression of toll-like receptor 4 and nucleotide-binding oligomerization domain-containing protein 1 in the jejunum, indicating that bacitracin reduced gram-negative pathogen invasion through the intestinal tract of nursery pigs (Table 2). The reduction of the expression of these receptors is primarily attributed to modulation of intestinal microbiota including both luminal and mucosa-associated microbiota by bacitracin, which is primarily due to positive modulation of jejunal mucosa-microbiota (Table 1 and Table 2). Subsequently, the decrease of these receptors reduce inflammation in the intestinal tract, as evidenced by decreased expression of tumor necrosis factor-alpha, IL-1β, and IL-6 in the small intestine (Table 1 and Table 2). The reduced inflammation in the intestinal tract also decreases oxidative stress (as indicated by lower levels of malondialdehyde and protein carbonyl), thereby improving the capacity for digestion and absorption of nutrients (increased villus height to crypt depth ratio and brush-border enzyme activity). The reduced inflammation in the intestinal tract caused by bacitracin or carbadox also increased the expression of tight junction proteins in the small intestine, thereby enhancing the integrity of the intestinal barrier. Another mechanism by which antimicrobial growth promoters modulate intestinal microbiota to improve intestinal health is through the reduction of toxic metabolites (e.g., ammonia, amines, phenols, indoles, etc.) in the intestinal tract [67,68,178,179]. Generally, toxic metabolites are produced by protein-fermenting bacteria, and these bacteria can be potentially suppressed by antimicrobial growth promoters. However, the effects of antimicrobial growth promoters on the concentration of toxic metabolites are inconsistent. For example, antimicrobial growth promoter cocktail (chlortetracycline, sulfamethazine, and penicillin) decreased urinary *p*-cresol concentration of pigs compared with pigs fed an antibiotic-free diet [180]. However, the combination of antimicrobial growth promoters (olaquindox, oxytetracycline calcium, and kitasamycin) increased the concentration of ammonia, phenol, *p*-cresol, indole, and skatole in the cecal digesta of pigs [181]. Based on the aforementioned mechanisms, the improvement of intestinal health through modulation of intestinal microbiota by antimicrobial growth promoters can enhance growth of pigs (Figure 6).

However, the modulation of intestinal microbiota by antimicrobial growth promoters may directly enhance pig growth without necessarily affecting intestinal health. To sustain maintenance and growth, both commensal and pathogenic intestinal microbiota require nutrients and compete with the host for nutrients [182]. For instance, microbiota in the small intestine utilize glucose, which accounts for 6% of the net energy in a pig diet [183]. However, antimicrobial growth promoters increase the likelihood of nutrients being absorbed in the small intestine by the host by suppressing overall microbial communities in the intestinal tract [184]. Increasing nutrient uptake by the host can potentially enhance the growth performance of pigs because the small intestine is the primary site for nutrient absorption [67]. Generally, antimicrobial growth promoters reduce the richness and abundance of intestinal microbiota, as indicated by a decrease in alpha diversity [185,186]. In contrast, the effects of bacitracin or carbadox on diversity metrics are inconsistent in the literature (Table 1 and Table 2), primarily due to differences in diet type, duration of feeding, and sample type [187]. Antimicrobial growth promoters may wipe out beneficial bacteria as well as pathogenic bacteria in the intestinal tract of pigs. For example, the supplementation of bacitracin or carbadox in pig diets decreased beneficial bacteria including *Prevotellaceae* nk3b31 (fiber utilizing bacteria) [188], *Peptococcus* (positively correlated with growth performance) [189], *Lactobacillaceae* (positively modulating immune system) [26], *Agathobacter* (butyrate-producing bacteria) [190], and *Megasphaera* (medium-chain fatty acid-producing bacteria; Table 1 and Table 2) [191]. Suppressing these beneficial bacteria might lead to reduction in energy provision and microbial dysbiosis. Specifically, reduction of the activity of fiber-utilizing or volatile fatty acid-producing bacteria by antimicrobial growth promoters potentially decreases fiber fermentation activity in the intestinal tract [192,193]. Subsequently, the production of volatile fatty acids from the microbial fermentation of fiber decreases, leading to a reduced contribution of energy to pigs from these fatty acids [192,194]. Moreover, volatile fatty acids can suppress the activity of pathogenic bacteria in the intestinal tract by decreasing intestinal pH [195]. An increase in intestinal pH may promote the proliferation of potentially pathogenic bacteria, leading to microbial dysbiosis [196].

**Table 2:** Effects of bacitracin on intestinal microbiota, intestinal health, and growth of nursery pigs. The ‘↑’ indicates an increase and the ‘↓’ indicates a decrease ^1^.

Initial BW, kg (Age, d)	Feeding Duration, d	Inclusion Rate, mg/kg	Sample Type	Alpha-/Beta- Diversity and Relative Abundance	Intestinal Health	Growth Performance	Reference
9.3 kg (d 28)	28	15	Cecal digesta	Beta diversity: *p* < 0.05 (Unweighted and Weighted UniFrac distance) *Prevotellaceae_NK3B31* ↓ *Lachnospiraceae_unclassified* ↓ *Streptococcus* ↓	Villus heigh to crypt depth in the ileum, 100% ↑	-	Lin and Yu [197]
9.0 kg (d 21)	42	15	Feces	Beta diversity: *p* < 0.05 (Weighted principal coordinate analysis) *Streptococcus* ↓ *Treponema 2* ↑ *Lachnospiraceae_unclassified* ↑	-	ADG, 7% ↑ ADFI, 6% ↑	Lin and Yu [198]
6.6 kg (d 21) *	28	30	Jejunal mucosa	Chao 1 ↑ Simpson ↑ Shannon ↑ *Acinetobacter* ↑ *Bifidobacterium* ↑ *Pseudomonas* ↑	Villus height in the jejunum, 22% ↑ Villus heigh to crypt depth in the jejunum, 30% ↑	ADG, 39% ↑ ADFI, 23% ↑ G:F, 10% ↑	Xu et al. [25]
6.3 kg (d 21) *	28	30	Feces	-	Protein carbonyl in the jejunum, 29% ↓	ADG, 22% ↑ ADFI, 12% ↑ G:F, 11% ↑	Duarte et al. [80]
			Jejunal mucosa	-			
6.6 kg (d 21)	30	150	Jejunum	Chao 1 ↓ Shannon ↓ Beta diversity: *p* < 0.05 (Bray-Curtis) *Clostridium_sensu_stricto* ↓ *Butyrivibrio* ↑	mRNA expression of aminopeptidase, maltase-glucoamylase, and sucrase-isomaltase in the jejunum ↑	ADG, 6% ↑ ADFI, 3% ↑ G:F, 3% ↑	Ángel-Isaza et al. [199]
7.9 kg (d 21) *	28	30	Jejunal mucosa	Simpson ↑ Shannon ↑ *Sphingomonadaceae* ↑ *Propionibacteriaceae* ↑	TNF-α in the jejunum, 22% ↓ Malondialdehyde in the jejunum, 46% ↓ mRNA expression of interferon-γ ↑ mRNA expression of TLR4 and NOD1 ↓	ADG, 12% ↑ ADFI, 6% ↑ G:F, 3% ↑	Duarte et al. [26]
9.9 kg (d 28)	28	30	Feces	Shannon ↓ Beta diversity: *p* < 0.05 (Unweighted and Weighted UniFrac distance) *[Ruminococcus] gauvreauii* ↓ *Ruminococcus UCG-005* ↓	-	ADG, 3% ↑ ADFI, 6% ↑ G:F, 2% ↓	Hung et al. [168]

* Asterisk indicates studies in which pigs were challenged with enterotoxigenic *E. coli*. ^1^ ADG = average daily gain; ADFI = average daily feed intake; G:F = gain to feed.

**Table 3:** Effects of carbadox on intestinal microbiota, intestinal health, and growth of nursery pigs. The ‘↑’ indicates an increase and the ‘↓’ indicates a decrease ^1^.

Initial BW, kg (Age, d)	Feeding Duration, d	Inclusion Rate, mg/kg	Sample Type	Alpha-/Beta- Diversity and Relative Abundance	Intestinal Health	Growth Performance	Reference
(d 21)	28	55 (first 14 d) 27.5 (last 14 d)	Feces	Chao 1 ↓ Shannon ↓ Faith’s phylogenetic diversity ↓ *Slackia* ↓ *Peptococcus* ↓ *Catenibacterium* ↓	-	-	Lourenco et al. [200]
5.8 kg (d 19)	33	55	Feces	*Veillonellaceae* ↑ *Streptococcus* ↓	-	-	Muurinen et al. [201]
6.9 kg *	12	0.5	Colonic digesta	*Lactobacillaceae* ↓	-	ADG, 17% ↓ ADFI, 7% ↓ G:F, 11% ↓	Kim et al. [202,203]
	12	50	Colonic digesta	*Lactobacillaceae* ↑	Villus heigh to crypt depth in the jejunum, 49% ↑	ADG, 17% ↑ ADFI, 5% ↑ G:F, 23% ↑	
	18	0.5	Colonic digesta	-	-	ADG, 6% ↓ ADFI, 5% ↓ G:F, 1% ↓	
	18	50	Colonic digesta	Beta diversity: *p* < 0.05 (Bray-Curtis) *Prevotellaceae* ↓ *Lactobacillaceae* ↓ *Clostridiaceae* ↑	Villus heigh to crypt depth in the jejunum, 35% ↑ Villus height in the jejunum, 20% ↑ mRNA expression of ZO-1 and occludin in the ileum ↑ mRNA expression of IL-1β, IL-6, and TNF-α in the ileum ↓	ADG, 14% ↑ G:F, 15% ↑	
6.2 kg (d 21) *	28	50	Jejunal digesta	*Streptococcus* ↑ *Bifidobacterium* ↓	Villus height in the jejunum, 25% ↑ mRNA expression of IL-6 in the ileum ↓	ADG, 53% ↑ ADFI, 35% ↑ G:F, 15% ↑	Jinno et al. [204] He et al. [177]
	28	50	Ileal digesta	Shannon ↑ *Lactobacillaceae* ↓ *Clostridium sensu stricto* 1 ↑			
	28	50	Colonic digesta	*Lactobacillaceae* ↓ *Streptococcus* ↓ *Clostridium sensu stricto* 1 ↑			
7.2 kg (d 21 to 24) *	12	50	Colonic digesta	*Streptococcaceae* ↓	mRNA expression of ZO-1 and occludin in the jejunum ↑ mRNA expression of IL-1β and IL-6 in the ileum ↓	ADG, 26% ↑ ADFI, 5% ↓ G:F, 33% ↑	Kim et al. [205,206]
	18	50	Colonic digesta	*Clostridiaceae* ↑ *Lactobacillaceae* ↓	mRNA expression of occludin in the jejunum ↑	ADG, 19% ↑ ADFI, 2% ↓ G:F, 16% ↑	
7.4 kg (d 21) *	7	50	Feces	*Blautia* ↑ *Escherichia-Shigella* ↓	*-*	ADG, 15% ↑ ADFI, 30% ↑ G:F, 12% ↓	Jinno et al. [207]
	14	50	Feces	*Agathobacter* ↓	*-*	ADG, 26% ↑ ADFI, 6% ↑ G:F, 19% ↑	
	21	50	Feces	*Dorea* ↓ *Streptococcus* ↓	*-*	ADG, 15% ↑ ADFI, 8% ↑ G:F, 6% ↑	
	28	50	Feces	*Blautia* ↓ *Dorea* ↓ *Lactobacillus* ↓	*-*	ADG, 16% ↑ ADFI, 15% ↑ G:F, 1% ↑	
	28	50	Ileal digesta	*Clostridium sensu stricto* 1 ↑ *Megasphaera* ↓	*-*	ADG, 16% ↑ ADFI, 15% ↑ G:F, 1% ↑	

* Asterisk indicates studies in which pigs were challenged with enterotoxigenic *E. coli*. ^1^ ADG = average daily gain; ADFI = average daily feed intake; G:F = gain to feed.

## 5. Impact of Antimicrobial Growth Promoter Alternatives on Intestinal Microbiota, Intestinal Health, and Growth of Pigs

Peer-reviewed papers on alternatives to antimicrobial growth promoters were searched from Scopus using the keywords ‘pig’ and each of alternatives for systemic review.

### 5.1. Organic Acid

Scientists outlined five key criteria to define a healthy gastrointestinal tract, which include efficient digestion and absorption of nutrients, the absence of gastrointestinal disorders, a balanced and stable intestinal microbiota, a robust and effective immune system, and an overall state of well-being [215]. Recent papers reported that intestinal microbiota have a direct or indirect connection with intestinal health of pigs and can be used as an indicator or biomarker for pigs [39,40]. Developing a robust microbiota early in life is essential for intestinal development and growth of pigs because it significantly influences intestinal function and enhances immune system maturation [216,217]. Organic acids, as a potential alternative to antimicrobial growth promoters, have been used in pig diets for decades because of their antimicrobial effects and reduction of pH in the gastrointestinal tract. The most recent research on organic acids and their effects on the modulation of intestinal microbiota, intestinal health, and growth performance of nursery pigs is listed in Table 4.

The effects of organic acids on diversity of microbiota are not consistent [218,219,220]. The changes of diversity are highly dependent on the sources of organic acids, dosage, and experimental animals. However, most studies showed an increased abundance of beneficial bacteria, such as *Lactobacillus* [219,221], whereas reduced abundance of potential pathogens, such as *E. coli* [222]. The effects of modulating intestinal microbiota by organic acids could contribute to the mechanisms of organic acids that were mentioned above, such as low cellular pH environment and the impact of anion on DNA replication. In addition, some of gram-positive bacteria (e.g., *Bacillus cereus*) are beneficial and possess a variety of acid resistance systems that enable them to overcome challenges of diverse acidic environments [215]. Interestingly, under the challenge model, some studies indicated that the inclusion of organic acids promoted balanced microbiota composition by reducing potentially harmful bacteria, such as *Enterobacteriaceae*. This effect partially substituted the use of traditional antimicrobial growth promoters in pig diets [223,224,225]. However, the results are inconsistent across previous studies. Pluske et al. [226] showed that a combination of formic acid, propionic acid, and phenolic compounds did not mitigate the negative impacts caused by F4 enterotoxigenic strain of *E. coli*. Therefore, the strain source of the challenge may be one of the factors contributing to the observed variability.

Modulation of intestinal microbiota by organic acids improves the intestinal health of nursery pigs. Most recent studies on organic acids focused on modulation of luminal microbiota (Table 4). Decreased relative abundance of pathogenic bacteria such as *E. coli* and *Enterobacteriaceae* in luminal digesta by organic acids may potentially improve antioxidant capacity of nursery pigs. Specifically, the supplementation of organic acids into pig diets decreased oxidative stress (lipid peroxidation), whereas, the increased expression of glutathione peroxidase and superoxide dismutase in the small intestine of nursery pigs (Table 4). Improved antioxidant capacity also benefits the increased villus height, expression of tight junction proteins, cytokines involved in combating bacterial infection (interferon-γ), mucin production (MUC2), and the production of mucin-secreting cells (goblet cells), which help protect the intestinal tract against pathogens.

In contrast to luminal microbiota which directly interact with digesta, mucosa-associated microbiota directly interact with intestinal immune cells [40,227]. Cytokines, chemokines, and metabolites produced by mucosa-associated microbiota are key molecular mediators of intestinal health which shape the responses of both the host and mucosa-associated microbiota [228]. Papadopoulos et al. [229] found that the supplementation of encapsulated organic acids modulated intestinal immunity by increasing neutrophil cells and Foxp3^+^ cells in jejunal mucosa of pigs. Grilli et al. [230] reported that the inclusion of mixture of citric acid, sorbic acid, thymol, and vanillin reduced the expression of immune-related genes, such as IL-6, IL-12, and TGF-β, in the ileal mucosa of pigs. Therefore, exploring changes in mucosa-associated microbiota presents an intriguing direction for future research.

The modulation of intestinal microbiota with organic acids may potentially improve pig growth through the mechanisms aforementioned. However, growth performance outcomes associated with dietary organic acid supplementation are not always consistent because they are strongly influenced by factors such as sources, dosage, duration of supplementation, and age or commensal microbiota of pigs. [221,224,231]. Benzoic acid, citric acid, fumaric acid, formic acid, and formate salts are commonly used in pig diets [232,233]. According to the recent review by Choi and Kim [93], the efficiency of organic acids in increase in body weight gain of nursery pigs was the most pronounced by benzoic acid (9.5% increase), followed by formic acid (7.1%), formate salts (6.9%), fumaric acid (5.0%), and citric acid (3.0%). This suggests that benzoic acid might be a potent organic acid for improving the growth of nursery pigs. Based on the recent meta-analysis [93], the optimal inclusion rate of benzoic acid in pig diets to maximize growth is 0.6%, but the supplementation of benzoic acid over 2.5% in pig diets could negatively affect growth of nursery pigs due to acidosis [233]. However, each organic acid has its own benefits. Citric acid is often used in drinking water supply as well as in pig diets because of its high solubility. However, 0.55 g/L of citric acid did not enhance growth performance or water consumption in nursery pigs, suggesting that a higher level may be needed to observe improvement [234]. Fumaric acid can improve energy and amino acid digestibility and inhibit pathogenic bacteria in the intestinal tract of pigs. However, the supplementation of fumaric acid is highly dependent on buffering capacity of diets and inclusion rate of fumaric acid [235]. Specifically, the beneficial effects on modulating intestinal microbiota and nutrient digestibility were observed only when pigs were fed diets with low buffering capacity, compared with diets containing high buffering capacity (with 3% sodium bicarbonate) [83]. Furthermore, increasing fumaric acid from 0 to 3% resulted in a linear decrease in lipopolysaccharide concentration in the digesta of pigs, suggesting that fumaric acid has antimicrobial effects [83,235]. Formic acid is very effective in reducing pH of the intestinal tract of pigs, which can inhibit potential pathogenic bacteria [236]. Recently, the salt form of formic acid (formate salt) has become commonly used because it is odorless and easier to process due to its lower volatility [237]. However, formic acid or formate salts should not exceed 1.2% in diets for nursery pigs and 1.8% in diets for growing pigs due to their negative impact on growth performance [238,239]. Therefore, the maximum allowable concentration of formic acid in pig diets within the European Union is 1.2% [240]. The combination of different organic acids may have synergistic effects on intestinal health and growth in pigs. For example, 0.9% of organic acid blend consisting of 75% formic acid and 25% propionic acid reduced the relative abundance of pathogenic bacteria in the intestinal tract of pigs challenged with *Salmonella* and *E. coli* [241]. The supplementation of 0.1% of organic acid blends including fumaric acid, citric acid, malic acid, capric acid, and caprylic acid improved weight gain and feed efficiency of nursery and growing-finishing pigs [242,243,244]. Similarly, the supplementation of 0.8 or 1.2% of organic acid blend (50% of lactic acid and 50% of formic acid) reduced *Salmonella* in mesenteric lymph nodes of finishing pigs [245].

### 5.2. Phytobiotics

Similar to organic acids, phytobiotics can positively modulate intestinal microbiota by promoting beneficial bacteria and inhibiting pathogenic bacteria in both luminal and mucosa-associated microbiota. The most recent research about probiotics and their effects on modulating intestinal microbiota, intestinal health, and growth performance of nursery pigs is listed in Table 5. For example, phytobiotics increased the relative abundance of beneficial bacteria (e.g., *Lactobacillus*, *Bifidobacterium*, *Lactococcus*) while decreasing the relative abundance of pathogenic bacteria (e.g., *Helicobacteraceae*, *Corynebacterium*, *E. coli*, *Clostridia*) in intestinal microbiota (Table 5). This modulation of the intestinal microbiota potentially reduces the expression of pattern recognition receptors (such as toll-like receptor 4 and NF-κB) in the small intestine, which reduces excessive inflammation, oxidative stress (evidenced by decreased protein carbonyl levels and increased glutathione peroxidase activity in the jejunum), and subsequently increases villus height in the small intestine (Table 5). In addition to reducing oxidative stress through the downregulation of pattern recognition receptors, the antioxidant properties of phytobiotics are partially attributed to their bioactive compounds, such as phenolics and flavonoids [246]. These compounds stabilize electrons from free radicals, thereby inhibiting radical chain reactions. Furthermore, phytobiotics enhance receptor-mediated phagocytosis and activate macrophages, leading to improved phagocytic and bactericidal activities of macrophages [247,248].

Phytobiotics can be classified into phenolic compounds, terpenes, and aldehydes based on their biosynthetic pathways [249]. Basil, clove, sage, and thyme are typical types of phenolic compounds, whereas terpenes include cinnamon, menta, and bluegum [77]. The most commonly used aldehydes are vanillin and cinnamaldehyde [77]. One of the most commonly used phytobiotics in pig diets are derived from oregano because of its distinct bioactive terpenes including carvacrol, thymol, γ-terpinene, and *p*-cymene [31,250]. According to the recent review [77], the growth-promoting effects of phytobiotics based on phenolic compounds (35% increase) were greater than those of phytobiotics based on terpenes (10% increase) or aldehydes (2% increase) in pigs.

As stated above, oregano extract is the one of the commonly used phytobiotics in pig diets. The positive effects of oregano extract on intestinal health were observed in nursery and growing pigs [31,251]. Furthermore, oregano extract reduces oxidative stress and stress associated with transportation in finishing pigs [252]. However, the effects of oregano extract on immune modulation are not transmitted from sows to nursery pigs. Supplementing 0.025% of oregano extract to sows during gestation and lactation did not affect the immune and growth responses of their nursery pigs [253]. Furthermore, there are concerns that supplementing oregano extract to finishing pigs may negatively impact the flavor of pork, thereby reducing its value [254]. Thyme is another commonly used phytobiotic in pig diets. Thyme is known for having one of the highest concentrations of antioxidant compounds among herbs [255]. Additionally, thyme exhibits anti-inflammatory properties due to its bioactive compounds, including phenolics such as rosmarinic acid and flavonoids like lutein, zeaxanthin, apigenin, luteolin, naringenin, and thymonin [256]. Supplementing 3% of thyme increased protein and fiber digestibility, villus height in the ileum, and plasma lysozyme and IgA concentrations in growing pigs [257]. Furthermore, the supplementation of 3% of thyme increased antioxidant activity and reduced oxidative stress in plasma and muscle of finishing pigs, indicating that thyme can improve health status and welfare of pigs [258]. These improvements contributed to enhanced weight gain, feed efficiency, and increased loin area. However, these improvements were not observed when pigs were fed 1% of thyme, indicating that high level of thyme may be required to expect these improvements. In addition, a mixture of thyme with different herbs (e.g., buckwheat, curcuma, black pepper, and ginger) is widely used in pig diets [259,260]. In contrast to oregano, thyme is more commonly used in growing-finishing pigs compared with nursery pigs because thyme can improve meat quality (e.g., increase in polyunsaturated fatty acid, loin muscle area, etc.).

Pig producers should be cautious about potential toxicity and anorexia of phytobiotics. In contrast to antimicrobial growth promoters and their alternatives, phytobiotics can potentially be detrimental to intestinal health and growth of nursery pigs. For example, Mo et al. [261] reported that supplementing 0.05% essential oil increased the expression of toll-like receptors 4 and 8, which increased the expression of pro-inflammatory cytokines (tumor necrosis factor-α and IL-1β). Certain compounds in essential oils such as thymol might trigger pro-inflammatory responses by inhibiting hydrolysis of ADP and deamination of adenosine in the extracellular environment of the intestine [262]. Furthermore, feeding essential oils or herbs may reduce feed intake which can decrease growth of pigs (Table 5) primarily due to their distinct odors and flavors [77]. However, issues related to toxicity or reduced appetite may be mitigated when phytobiotics are encapsulated, as microencapsulation slows their release and makes them odorless [263].

### 5.3. Probiotics

Another alternative to antimicrobial growth promoters, probiotics, have been widely used in pig production because of their beneficial effects on intestinal microbiota, intestinal health, and growth of pigs. However, the beneficial effects of probiotics are highly strain specific [264]. The selection and use of probiotics are mostly based on empirical experience [265]. Therefore, selection of probiotics is a critical process and should involve consideration of the following characteristics: colonization ability, health-promoting effects, applicability, and safety [266,267].

Probiotics are commonly used in pig diets, particularly for nursery pigs, because their immune system and intestinal microbiota are not yet fully developed [268,269]. The most recent research about probiotics and their effects on modulating intestinal microbiota, intestinal health, and growth performance of nursery pigs is listed in Table 6. Most studies showed that intestinal microbiota composition and alpha diversity changed after supplementing probiotics in pig diets [270,271,272,273,274,275]. However, the supplementation of probiotics in pig diets did not consistently increase the relative abundance of the strain of the probiotics [270,271,272,273,274,276,277]. Rather, probiotics containing lactic acid-producing bacteria, such as *Bacillus*, *Lactobacillus*, *Bifidobacterium*, and *Enterococcus*, can increase the relative abundance of other lactic acid-producing bacteria in the intestinal microbiota, even if the specific bacterial strains from probiotics are not identical [277,278]. This cross-support likely arises from their ability to create a favorable environment by producing lactic acid, which suppresses pathogenic bacteria and fosters growth of other beneficial microbes in the intestinal tract [279]. This positive modulation of the intestinal microbiota by organic acids is expected to decrease endotoxins secreted by pathogenic bacteria (e.g., *E. coli*, *Campylobacter*, etc.) and reduce the expression of pattern recognition receptors including toll-like receptor 4 in the small intestine (Table 6). Subsequently, inflammatory immune responses can be reduced (as evidenced by reduced IL-1β, IL-6, tumor necrosis factor alpha-α, NF-κB). Interestingly, probiotics are expected to reduce apoptosis of intestinal cells because NF-κB also promotes apoptosis of cells [280]. This hypothesis aligns with the increased expression of proliferating cell nuclear antigen in the small intestine by probiotics (Table 6), which helps repair enterocytes [281]. This hypothesis aligns with the increased expression of proliferating cell nuclear antigen in the reduced apoptosis of cells and increased cell proliferation in the small intestine due to probiotics being closely associated with the improvement of intestinal morphology, including increased villus height and goblet cell numbers.

The majority of studies about probiotics focused on luminal microbiota rather than mucosa-associated microbiota. As mentioned above, mucosa-associated microbiota, intestinal epithelial cells, and intestinal immune cells engage in intricate interactions, creating a dynamic and finely balanced system. This interplay is essential for maintaining nutrition and immune functions of the intestinal tract, ensuring effective nutrient absorption while defending against pathogens through immune regulation and epithelial barrier integrity [228,282]. This makes mucosa-associated microbiota another essential indicator to evaluate the effect of probiotics on intestinal health of pigs [283].

The modulation of intestinal health by probiotics, based on the aforementioned mechanisms, is expected to be closely linked to increased growth in pigs. In addition, energy and nutrient digestibility are one of the keys to growth of pigs. Generally, pigs with healthy intestinal status have greater energy and nutrient digestibility than pigs with poor intestinal status [284]. The efficacy of different probiotics on energy and nutrient digestibility may be different. For example, *Bacillus* and *Lactobacillus* are the most commonly used probiotic strains in pig diets because these bacteria are primary beneficial bacteria in the intestinal tract of pigs [285,286]. Based on our recent analysis, the increase in energy and protein digestibility was similar between *Bacillus*-based probiotics (3.2% and 2.0%, respectively) and *Lactobacillus*-based probiotics (1.6% and 1.0%, respectively). However, it is challenging to identify a specific response criterion in intestinal health that is solely responsible for improving growth. Moreover, the increase in the relative abundance of specific beneficial bacteria in the intestinal tract may not be the sole factor responsible for the improvement in pig growth [287,288]. Similar to organic acids, the efficacy of probiotics can be enhanced by increasing the substrate available for probiotics [289,290], providing protective coatings [291], and using combinations of different probiotic strains [292]. Additionally, multi-strain probiotics may have a greater impact on intestinal health and growth in pigs compared with single-strain probiotics because of expected synergistic effects of different strains. However, the effects of multi-strain probiotics on growth of pigs are not consistent. For example, the supplementation of both *Bacillus subtilis* and *Bacillus licheniformis* increased average daily gain by 16% compared with the control [293]. However, the combination of *Bacillus subtilis* and *Bacillus amyloliquefaciens* did not improve growth of pigs in other studies [294,295]. Similarly, the combination of *Lactobacillus acidophilus*, *Bacillus subtilis*, and *Saccharomyces cerevisiae* improved the average daily gain of pigs by 12% and 6% [296,297], respectively, but did not affect average daily gain in another study [37]. The inconsistency in the effects of multi-strain probiotics is attributed to factors such as the inclusion rate, probiotic formulation, nutritional density of feeds, and the health status of pigs. However, challenges associated with using probiotics in pig production exist. For example, probiotics may exhibit pathogenicity in the intestinal tract of pigs because selection of probiotic strains generally focuses on enhancing their ability to adhere to the intestinal lining [298]. Furthermore, the immunomodulatory effects of probiotics may either excessively stimulate or suppress the immune system of pigs [265,298].

**Table 4:** Effects of organic acid on intestinal microbiota, intestinal health, and growth of nursery pigs. The ‘↑’ indicates an increase and the ‘↓’ indicates a decrease ^1^.

Type	Initial BW, kg	Feeding Duration, d	Inclusion Rate, %	Sample Type	Alpha-/Beta- Diversity and Relative Abundance	Intestinal Health	Growth Performance	Reference
Protected sodium butyrate	6.5	39	0.3, 0.2, 0.1 in phase 1, 2, 3, respectively	Feces	*Escherichia coli* ↓ Total coliforms ↓	Lipid peroxidation in the jejunum, 19% ↓ Glutathione peroxidase in the jejunum, 58% ↑ Superoxide dismutase, 58% ↑	ADG, 2% ↓ ADFI, 7% ↓ G:F, 3% ↑	Marchiori et al. [222]
Tributyrin	6.5	39	0.3, 0.2, 0.1 in phase 1, 2, 3, respectively	Feces	*Escherichia coli* ↓ Total coliforms ↓	Lipid peroxidation in the jejunum, 38% ↓ Superoxide dismutase, 46% ↑	ADG, 7% ↑ ADFI, 3% ↓ G:F, 10% ↑	
Lauric acid, butyrate, medium-chain fatty acids	7.4	42	0.2	Feces	Spirochaetes ↓	mRNA expression of superoxide dismutase 1, glutathione peroxidase 1, and ZO-1 in the jejunum ↑ Villus height to crypt depth ratio in the jejunum, 31% ↑	ADG, 13% ↑ ADFI, 10% ↑ G:F, 4% ↑	Cai et al. [218]
Sodium butyrate	5.9	14	0.1	Colonic digesta	*Lactobacillus* ↑ *Enterobacteriaceae* ↓ *Escherichia coli* ↓	Goblet cells in the ileum, 26% ↑	ADG, 3% ↓ ADFI, 9% ↓ G:F, 11% ↑	Sadurni et al. [221]
Gluconic acid	8.2	42	1.8	Distal small intestinal/Colonic digesta	Chao1 ↓ Simpson ↓ *Lactobacillus amylovorus* ↑ *Faecalibacterium prausnitzii* ↑ *Megasphaera elsdenii* ↑	Butyrate concentration in the cecum and colon ↑ mRNA expression of MUC2 and IFN-γ in the ileum ↑	ADG, 7% ↑ ADFI, 11% ↑ G:F, 1% ↓	Michiels et al. [219]
Encapsulated sodium butyrate	4.7	49	0.20, 0.15, 0.10 in phase 1, 2, 3, respectively	Cecal digesta	*Streptococcaceae* ↓	-	ADG, 5% ↑ ADFI, 1% ↓ G:F, 6% ↑	da Silva et al. [299]
Formic acid, ammonium formate, acetic acid	5.3	49	0.2	Cecal digesta	Beta-diversity: *p* < 0.05 (Jaccard distances) *Coprococcus* ↑ *Blautia* ↑	-	ADG, 4% ↑ ADFI, 3% ↑ G:F, 1% ↑	Xiang et al. [220]
Sodium butyrate, benzoic acid	6.9	35	0.105 sodium butyrate, 0.5 benzoic acid	Feces	Shannon ↑ Beta-diversity: *p* < 0.05 (Bray-Curtis) *Veillonella* ↓ *Sarcina* ↓ *Turicibacter* ↑	-	ADG, 3% ↓ ADFI, 11% ↓ G:F, 8% ↑	Wei et al. [300]
Sorbic acid, medium chain fatty acid, formic acid, short chain fatty acid	6.7	20	0.2 Presan FX and 0.3 Fysal MP	Feces	*Ruminococcaceae* ↑ *Lachnospiraceae* ↑ *Lactobacillaceae* ↑	-	ADG, 10% ↑ ADFI, 1% ↑ G:F, 5% ↑	Pluske et al. [226]
Formic acid, ammonium formate, propionic acid, acetic acid, citric acid	7.8	28	0.3	Cecal digesta	*Lachnospiraceae* ↓ *Escherichia-Shigella* ↓	mRNA expression of claudin-1 and ZO-1 ↑ Acetic acid concentration in the cecum, 29% ↑	ADG, 9% ↑ ADFI, 5% ↓ G:F, 14% ↑	Ma et al. [301]
Short chain fatty acid	8.7	42	0.2	Feces	*Clostridium sensu stricto 1* ↑ *Streptococcus* ↓	-	ADG, 4% ↑ ADFI, 2% ↑ G:F, 2% ↑	Lingbeek et al. [302]

^1^ ADG = average daily gain; ADFI = average daily feed intake; G:F = gain to feed.

**Table 5:** Effects of phytobiotics on intestinal microbiota, intestinal health, and growth of nursery pigs. The ‘↑’ indicates an increase and the ‘↓’ indicates a decrease ^1^.

Type	Initial BW, kg	Feeding Duration, d	Inclusion Rate, %	Sample Type	Alpha-/Beta- Diversity and Relative Abundance	Intestinal Health	Growth Performance	Reference
Mixture of castor oil and cashew nutshell liquid	7.0	34	0.50, 0.75, 1.00, or 1.50	Jejunal mucosa	*Helicobacteraceae* ↓ *Lactobacillus kitasatonis* ↑	Protein carbonyl in the jejunal mucosa ↓ Villus height in the jejunum ↑	-	Moita et al. [303]
Herb	6.4	28	1	Jejunal mucosa	Chao1 ↓ Shannon ↓ Simpson ↓	Protein carbonyl in the jejunum, 44% ↓ Villus height to crypt depth ratio in the jejunum, 35% ↑ Ki-67^+^ in the jejunum, 28% ↓	ADG, 13% ↓ ADFI, 13% ↓	Garavito-Duarte et al. [78]
Essential oil	6.4	28	1	Jejunal mucosa	*Syntrophococcus* ↓ *Corynebacterium* ↓	Villus height to crypt depth ratio in the jejunum, 29% ↑ Ki-67^+^ in the jejunum, 21% ↓	ADG, 2% ↑ ADFI, 2% ↓	
Essential oil	6.3	28	0.05	Cecal digesta	-	Glutathione peroxidase in the jejunum, 13% ↑ mRNA expression of TLR4 (460% ↑), TLR8 (1455% ↑), TNF-α (161% ↑), and IL-1β (366% ↑) in the ileum	ADFI, 13% ↓ G:F, 4% ↓	Mo et al. [261]
Microencapsulated essential oil	6.3	28	0.05	Cecal digesta	8 potential pathogenic bacteria ↓	Glutathione peroxidase in the jejunum, 30% ↑	ADG, 17% ↑ ADFI, 4% ↓ G:F, 2% ↑	
Essential oil	7.6	28	0.04	Colonic digesta	*Holdemanella* ↑ *phascolarctobacterium* ↑	Villus height in the ileum ↑ Expression of TLR4 and NF-κB in the ileum ↓	ADG, 27% ↑ ADFI, 25% ↑	Shao et al. [304]
Herbal plant extract	8.7	33	0.5	Feces	*E. coli* ↓ *Lactobacillus* ↑ *Bifidobacterium* ↑	Villus height in the ileum, 80% ↑	-	Shuo et al. [305]
Tannin	8.6	21	0.15	Colonic digesta	*Clostridium_sp_Culture_27* ↓ *Lactococcus* ↑	The activity of maltase and sucrase in the jejunum ↑	ADG, 22% ↑ ADFI, 10% ↑ G:F, 11% ↑	Xu et al. [306]
Tannic acid	7.7	28	0.1	Colonic digesta	*E. coli* ↓	Butyrate concentration in cecal digesta, 98% ↑ Villus height to crypt depth ratio in the ileum, 20% ↑	-	Song et al. [307]

^1^ ADG = average daily gain; ADFI = average daily feed intake; G:F = gain to feed.

**Table 6:** Effects of probiotics on intestinal microbiota, intestinal health, and growth of nursery pigs. The ‘↑’ indicates an increase and the ‘↓’ indicates a decrease ^1^.

Type	Initial BW, kg	Feeding Duration, d	Inclusion Rate (%), Daily Oral Administration (CFU/d), or Concentration of Probiotics in Diet (CFU/kg)	Sample Type	Alpha-/Beta- Diversity and Relative Abundance	Intestinal Health	Growth Performance	Reference
*Lactobacillus*	6.1	47	0.1%	Feces	*C. Incertae Sedis* XIII ↑	Villus height to crypt depth ratio in the jejunum, 5% ↑	ADG, 1% ↓ ADFI, 1% ↓ G:F, 2% ↓	Zuniga et al. [276]
*Bifidobacterium*	6.1	47	0.1%	Feces	*Streptococcaceae* ↓	Villus height to crypt depth ratio in the jejunum, 19% ↑	ADFI, 3% ↓ G:F, 1% ↓	
*Enterococcus hirae*	6.4	21	1.0 × 10^10^ CFU/d	Colonic digesta	Chao1 ↑ Simpson ↑ Channon ↑ Beta-diversity: *p* < 0.05 *Prevotellaceae* ↑ *Lactobacillaceae* ↓ *Bacteroidaceae* ↑	Acetic acid concentration in the colon ↑ mRNA expression of proliferating cell nuclear antigen and villus height in the jejunum ↑	ADG, 14% ↑ G:F, 11% ↓	Zhang et al. [270]
*Lactiplantibacillus argentoratensis*	5.9	24	1.0 × 10^8^ CFU/d	Feces	Beta-diversity: *p* < 0.05 (UniFrac distances) *Streptococcus* ↑ *Clostridium* ↑ *Campylobacter* ↓	-	ADG, 36 folds ↑	Yoon et al. [271]
*Bacillus*	8.1	31	0.04%	Feces	*Streptococcus* ↓ *Lactobacillus* ↑	Goblet cells in the ileum, 71% ↑ mRNA expression of TNF-α (81% ↓) and occludin (147% ↑) in the ileum	ADG, 5% ↑ ADFI, 2% ↑ G:F, 2% ↑	Xue et al. [278]
*Bacillus licheniformis*	8.2	10	2.5 × 10^9^ CFU/kg	Ileal digesta Colonic digesta	Bacteroidetes ↓	mRNA expression of ZO-1 (133% ↑), occludin (156% ↑), SGLT1 (202% ↑), and aminopeptidase N (98% ↑) in the ileum	ADG, 27% ↑ ADFI, 3% ↓ G:F, 26% ↑	Xu et al. [308]
*Lactobacillus plantarum*, *lactobacillus reuteri*, *bifidobacterium longum*	7.1	14	1.0 × 10^9^ CFU/kg	Feces	Beta-diversity: *p* < 0.05 (Bray–Curtis) *Faecalibacterium* ↑ *Parabacteroides* ↑ *Clostridium* ↑	mRNA expression of IL-1β, IL-6, TNF-α, and NF-κB in the duodenum ↓	ADG, 15% ↑	Tang et al. [272]
*Enterococcus faecium, bacillus subtilis*, and *saccharomyces cerevisiae*	13.1	42	0.5%	Feces	Shannon ↑ Beta-diversity: *p* < 0.05 (Bray–Curtis) *Ruminococcaceae* ↑ *Prevotella* ↑ *Eubaterium coprostanoligenes* ↑	-	ADG, 19% ↑	Park et al. [273]
*Clostridium butyricum*	8.2	28	1.5 × 10^9^ CFU/d	Feces	Beta-diversity: *p* < 0.05 (unifrac distance) *Faecalibacterium* ↑ *Rikenellaceae* ↓	-	ADG, 25% ↑ ADFI, 7% ↑ G:F, 19% ↑	Liu et al. [274]
*Lactiplantibacillus plantarum*, *Bacillus subtilis*	8.9	50	1.0 × 10^9^ CFU/kg	Feces	Shannon ↑ Beta-diversity: *p* < 0.05 (NMDS) *Lactobacillus* ↑ *Streptococcus* ↓ *Clostridium* ↓	-	ADG, 15% ↑ ADFI, 9% ↑ G:F, 4% ↑	Chen et al. [275]
*Bacillus licheniformis*	6.5	14	0.1%	Cecal digesta	*Lactobacillus* ↑ *Clostridium* ↓	Lipase activity, occludin, ZO-1, and villus height to crypt depth ratio in the jejunum ↑ Lactic acid concentration in the cecum, 212% ↑	ADG, 25% ↑ ADFI, 19% ↑ G:F, 7% ↑	Sun et al. [277]
*Bacillus subtilis*	6.4	28	2.0 × 10^9^ CFU/d	Feces	*Escherichia coli* ↓ Total coliforms ↓ *Bacillus* spp. ↑	mRNA expression of occludin and proliferating cell nuclear antigen in the ileum ↑ mRNA expression of TLR-4 in the ileum ↓	ADFI, 9% ↓ G:F, 13% ↑	Sudan et al. [309]

^1^ ADG = average daily gain; ADFI = average daily feed intake; G:F = gain to feed.

### 5.4. Other Potentials: Postbiotics, Functional Fatty Acid, and Bacteriophage

Postbiotics potentially protect intestinal linings from exogenous pathogens. For example, *Saccharomyces* yeast increased the expression of mTOR and interferon-γ, whereas decreased the expression of B-cell lymphoma 2-associated X protein 1 (BAX1) in the jejunum when nursery pigs were challenged with F18^+^ *E. coli* [28,29]. This result indicates that *Saccharomyces* yeast promotes proliferation of epithelial cells in intestinal tract (mTOR) [29,135], enhances the immune response against bacterial infections (interferon-γ) [135], and reduces apoptosis of epithelial cells (BAX1) [310] in the small intestine. The increased expression of mTOR and reduced BAX1 may lead to enhanced cell proliferation (Ki67^+^) and increased villus height in the jejunum, contributing to the growth of pigs. Furthermore, *Lactobacillus* postbiotics and *Saccharomyces* yeast positively modulate the intestinal microbiota, downregulating pattern recognition receptors (e.g., toll-like receptor 4, nucleotide-binding oligomerization domain-containing protein 1) and reducing inflammation and oxidative stress in the small intestine, which also contributes to the improvement of growth [25,26,29]. Yeast is the most frequently used postbiotic in pig diets due to the distinct components in its cell walls. For example, yeast cell walls consist of β-glucan, mannoprotein, and chitin, which can enhance intestinal health of pigs [311]. However, our recent analysis showed that yeast did not significantly increase energy and protein digestibility in pigs (0.9 and 0.8%, respectively), suggesting that improved digestibility may not contribute to enhanced growth when yeast is supplemented with pig diets.

Medium-chain (caproic acid, caprylic acid, capric acid, and lauric acid) and long-chain fatty acids (e.g., myristic acid) reduce the proliferation of pathogenic bacteria such as *Clostrium perfringens*, *Staphylococcus aureus*, *Helicobacter rappini*, *E. coli*, and *Streptococcus agalactiae*, whereas promote beneficial bacteria including *Bifidobacterium*, *Lactobacillus*, and *Megasphaera* in the intestinal tract of pigs [139,312,313,314,315,316,317,318,319]. This positive modulation of luminal or mucosa-associated microbiota potentially reduces inflammation and humoral immune responses (as evidenced by reduced expression of pro-inflammatory cytokines, Toll-like receptor 4, nucleotide-binding oligomerization domain 2, and intraepithelial lymphocytes in the small intestine), thereby increasing villus height in the jejunum and redirecting energy from immune functions to support growth. Caprylic acid, capric acid, and lauric acid have greater antimicrobial effects on pathogenic bacteria compared with other fatty acids [138]. Coconut oil and palm kernel oil are rich in caprylic acid, capric acid, and lauric acid. The blend of caprylic acid and capric acid is commonly used in pig diets rather than individually due to their synergistic effects on antimicrobial effects against pathogenic bacteria [320]. However, the antimicrobial effects of the blend of caprylic acid and capric acid are mainly observed on gram-positive bacteria rather than gram-negative bacteria. In contrast to caprylic acid and capric acid, lauric acid is often used individually in pig diets. The antimicrobial effects of lauric acid are similar to the blend of caprylic acid and capric acid. However, lauric acid may reduce feed intake in pigs by increasing the transit time of digesta in the intestinal tract and promoting the secretion of appetite-regulating hormones (e.g., CCK, PYY) [321]. Despite this reduction in feed intake, lauric acid can potentially improve gain to feed ratio without compromising growth of pigs [322]. However, the inclusion rate of lauric acid should not exceed 2.5% of pig diets based on cost-effectiveness analysis.

The efficacy of functional fatty acids may be improved when they are components of monoglycerides (glycerol + a single functional fatty acid) [138]. The potential advantage of monoglycerides over functional fatty acids is due to their ability to form micelles at lower concentrations compared with fatty acids, which aids in disrupting the phospholipid membranes of bacteria [323]. Furthermore, encapsulating or coating functional fatty acids can enhance their efficacy by slowing their absorption in the intestinal tract [324].

Unlike other antimicrobial growth promoter alternatives, bacteriophages have host specificity [325]. Generally, bacteriophage cocktails consist of bacteriophages targeting different pathogenic bacteria to which pigs are susceptible. For example, most of the bacteriophages used in previous pig studies contained *E. coli*, *Clostridium perfringens*, *Salmonella*, or *Staphylococcus aureus* [37,38,153,326,327]. Bacteriophage cocktails decreased the abundance of their target bacteria (*Clostridium* spp.) in cecal digesta and feces of nursery pigs as expected [37,153,326]. However, the bacteriophage cocktails also reduced the abundance of untargeted pathogenic bacteria (Coliform and *Escherichia-Shigella*), whereas increased the abundance of beneficial bacteria (*Lactobacillus* spp., *Bifidobacterium* spp., *Lactobacilaceae*, and *Eubacterium*) in digesta or feces of nursery pigs, and the reason for this remains unclear [37,38,153,326,327]. The suppression of pathogenic bacteria and the increased proliferation of beneficial bacteria may potentially enhance villus height in the small intestine, which in turn can improve nutrient digestibility of pigs, as demonstrated in previous studies on bacteriophages [37,38,153,326]. Furthermore, bacteriophages can also improve the integrity of tight junction proteins in the small intestine by inhibiting pathogenic bacteria that damage intestinal integrity, which contributes to improved growth of pigs [325]. The supplementation of bacteriophages targeting *E. coli*, *Clostridium perfringens*, *Salmonella*, or *Staphylococcus aureus* resulted in an average increase of 8.0% in body weight gain and 7.5% in gain to feed ratio of nursery pigs [37,38,153,326,327].

## 6. Comparison of Antimicrobial Growth Promoters and Their Alternatives and Future Research

Antimicrobial growth promoters (bacitracin and carbadox) and their alternatives (organic acid, phytobiotics, probiotics, postbiotics, functional fatty acids, and bacteriophages) modulate intestinal microbiota of pigs through distinct mechanisms. Despite their different mechanisms, antimicrobial growth promoters and their alternatives positively modulate luminal or mucosa-associated microbiota in most cases by promoting beneficial bacteria and suppressing potentially pathogenic bacteria (Table 1, Table 2, Table 3, Table 4 and Table 5). However, antimicrobial growth promoters potentially wipe out beneficial bacteria as well as pathogenic bacteria in the intestinal tract of pigs, which might cause dysbiosis of intestinal microbiota. In contrast to antimicrobial growth promoters, bacteriophages are designed to target only specific pathogenic bacteria and should not directly kill beneficial bacteria.

In general, the positive modulation of intestinal microbiota by antimicrobial growth promoters or their alternatives enhances intestinal health in pigs by reducing the expression of pattern recognition receptors, which decreases immune responses and inflammation in the intestinal tract. This reduction in immune responses and inflammation helps conserve energy and nutrients that would otherwise be used for the metabolic costs of activating immune functions. Furthermore, reducing intestinal inflammation decreases oxidative stress and enhances antioxidant activity, leading to improved villus height and repair of intestinal tissues. Improvement in intestinal health is attributed to enhanced growth in pigs. However, the effects of alternatives to antimicrobial growth promoters on growth performance of pigs show greater variability compared with antimicrobial growth promoters (Table 1, Table 2, Table 3, Table 4 and Table 5). This variability may be due to differences in inclusion rates and different sources within each alternative category.

The efficacy of alternatives to antimicrobial growth promoters can be improved by advanced technologies. For example, coating or microencapsulation can improve the efficacy of organic acids, phytobiotics, and functional fatty acids by gradually releasing them in the lower intestinal tract of pigs or by reducing their distinct odor and flavor. Moreover, probiotics can be modified to enhance their resistance to gastric acids, bile acids, and digestive enzymes, ensuring successful passage from pig diets to the intestinal tract. In addition, the efficiency and host range of bacteriophages can be enhanced through phage engineering.

Enzymes can serve as alternatives to antimicrobial growth promoters by enhancing intestinal health and nutrient digestibility, which helps mitigate antimicrobial resistance. Lysozyme is an enzyme that breaks glycosidic linkages in the peptidoglycan of bacterial cell walls and enhances immune response by stimulating IgA secretion and macrophage activation [328]. Lysozyme is particularly effective when pigs face immune challenges (e.g., *E. coli*, unsanitary environments) by reducing inflammatory responses in the intestinal tract and serum, while increasing villus height in the small intestine, thereby alleviating the negative impact [329,330]. Supplementing pig diets with xylanase or mannanase can hydrolyze non-starch polysaccharides such as xylan or mannan, allowing the release of entrapped nutrients and increasing nutrient digestibility [331,332]. Additionally, hydrolysis of xylan and mannan produces xylo-oligosaccharides and mannan-oligosaccharides, which exert prebiotic effects [40]. Furthermore, breaking down these non-starch polysaccharides reduces digesta viscosity and increases short-chain fatty acid in the intestinal tract, enhancing growth performance [332]. For example, supplementing xylanase positively influenced mucosa-associated microbiota in the intestinal tract, reduced digesta viscosity, plasma tumor necrosis factor-α, and jejunal oxidative stress, and enhanced energy digestibility, contributing to improved growth of pigs [333,334]. Similar to xylanase, supplementing mannanase reduced digesta viscosity, intestinal inflammation (tumor necrosis factor-α and IgG), and oxidative stress while increasing nutrient digestibility, villus height, tight junction proteins, and beneficial bacteria such as *Lactobacillus* in the intestinal tract, leading to improved pig growth performance [332,335,336]. Furthermore, supplementing various enzymes in pig diets is common because each enzyme targets different substrates, and hydrolyzing these substrates can have synergistic effects on intestinal health and pig growth. For example, xylanase is often supplemented with mannanase, glucanase, protease, or phytase in pig diets [13,337,338,339].A combination of different antimicrobial growth promoters may have synergistic effects on intestinal health and growth of pigs. For instance, combining organic acids with essential oils enhances tight junction proteins and promotes the growth of beneficial bacteria such as *Lactobacillus* in intestinal tract of pigs [340]. Positive synergistic effects between probiotics (*E. faecium* DSM 7134 or *E. faecium* SF68) and medium-chain fatty acids (caproic acid, caprylic acid, capric acid, and lauric acid) or organic acids (benzoic acid) were observed in nitrogen digestibility of pigs [341,342]. Potential synergistic effects of combining three alternatives to antimicrobial growth promoters (organic acids medium-chain fatty acids and probiotics) were reported on growth and nitrogen digestibility in pigs, suggesting benefits for growth and intestinal health [343]. Therefore, further research is needed to determine the optimal combination of alternatives to antimicrobial growth promoters for maximizing intestinal health and growth in pigs.

One of the reasons for replacing antimicrobial growth promoters in pig diets with their alternatives is to minimize antibiotic resistance in intestinal tract. However, bacteria in the intestinal tract may develop resistance to alternatives to antimicrobial growth promoters in different ways that are not fully understood, requiring further research to clarify. For example, acid-sensitive bacteria such as *Salmonella* or *E. coli* in intestinal tract or meat products exposed to moderately low pH (pH 5.8) by weak acids such as acetic acid or propionic acid can synthesize acid shock proteins which can protect bacteria from extremely acidic conditions (pH 3.0) [344,345]. Bacterial resistance to phytobiotics has not been reported. Some probiotics based on *Lactobacillus* or *Bifidobacterium* carry antibiotic-resistance genes and transfer them to another bacteria [346]. For example, *Lactobacillus*-based probiotics can transfer vancomycin-resistant gene plasmids to *E. faecalis* in mice [347]. A mutation of single gene (for reducing affinity of erythromycin for ribosome) occurs in *Lactobacillus rhamnosus*, which induces resistance to macrolide [348]. Horizontal transfer of antibiotic resistance genes may occur in *Lactobacillus casei*, *Lactobacillus acidophilus*, *Lactobacillus reuteri*, *Lactobacillus rhamnosus*, and *Lactobacillus delbrueckii* [349]. In contrast to *Lactobacillus*, *Bifidobacterium* is intrinsically resistant to a few antibiotics [350,351] (e.g., mupirocin and aminoglycoside). *Bifidobacterium* develops antimicrobial resistance through gene modifications involved in antibiotic adenylation, ribosomal protection, antibiotic hydrolysis, and antibiotic acetylation [349]. Gram-negative bacteria including *E. coli* or *Salmonella typhimurium* can develop resistance to medium-chain fatty acids including capric acid by blocking their passage through cell membranes [352]. Furthermore, bacteria can resist medium-chain fatty acids by metabolizing them through β-oxidation [353]. Resistance to bacteriophage has not been reported in farm animals. In humans, the rate of developing resistance to bacteriophages is about 10 times lower than that of antibiotics [354]. Furthermore, using a bacteriophage cocktail instead of an individual bacteriophage can delay the emergence of bacteriophage-resistant bacteria [355].

## 7. Conclusions

This review provided a comprehensive summary of the mechanisms and effects of antimicrobial growth promoters and their alternatives on intestinal microbiota of pigs. Furthermore, this paper summarized recent publications on how bacitracin, carbadox, organic acids, phytobiotics, probiotics, functional fatty acids, or bacteriophages modulate intestinal microbiota, intestinal health, and growth of pigs. The mechanisms of modulating intestinal microbiota vary depending on the type of antimicrobial growth promoters or their alternatives. Understanding the mechanisms of antimicrobial growth promoters and their alternatives is essential for developing novel alternatives. Furthermore, this review discussed how the modulation of intestinal microbiota affects intestinal health and, subsequently, growth of pigs. In addition, mucosa-associated microbiota may be an essential indicator for estimating intestinal health and growth of pigs because of their direct interaction with immune cells. However, the effects of antimicrobial growth promoters and their alternatives on intestinal microbiota, intestinal health, and growth performance are not consistent across different studies. Furthermore, the growth-promoting effects of alternatives to antimicrobial growth promoters may not be as consistent or pronounced as those of antimicrobial growth promoters. The efficacy of these alternatives can potentially be enhanced through advanced technologies such as coating or microencapsulation. Therefore, pig producers should carefully assess which growth promoter alternative is the most effective for optimizing both profitability and the health status of pigs in their production system. The decision should also consider the mechanisms of alternatives on intestinal microbiota, intestinal health, growth, and the findings reported in previous studies.

## Figures and Tables

**Table 1:** Summary of mechanisms of antibiotics.

Main Mechanism	Specific Mechanism	Example
Inhibition of cell wall synthesis	Inhibition of the cytoplasmic stage	Mur enzymes, alanine racemase, D-alanyl-D-alanine ligase, fosfomycin, and seromycin
	Inhibition of the membrane-associated stage	Tunicamycin, liposidomycin, mureidomycin, mannopeptimycin, lantibiotic, defensin, and glycopeptide antibiotics
	Inhibition of the extra-cytoplasmic stage	Transpeptidase, endopeptidase, carboxypeptidase, transglycosylase, penicillin, cephalosporin, and cephamycin
Disintegration of cell membrane	Formation of pores that disrupt the integrity of the cell membrane	Monensin, salinomycin, narasin, and nisin
Inhibition of protein synthesis	Interference with the aminoacyl site on the 30S subunit	Neomycin, kanamycin, puromycin, and tetracycline
	Interference with peptidyl transferase center on the 50S subunit	Chloramphenicol, clindamycin, sparsomycin, streptogramin, and oxazolidinone
	Interference with the exit site on the 50S subunit	Macrolide antibiotics
Inhibition of DNA synthesis	Inhibition of RNA transcription	Rifamycin
	Inhibition of Type II topoisomerases	Olaquindox, mequindox, quincetone, cyadox, and carbadox

## Data Availability

The data presented in this study are available upon request from the corresponding author.
